# A machine learning model for predicting cognitive improvement after scalp cluster needling therapy in patients with post-stroke mild cognitive impairment

**DOI:** 10.3389/fneur.2026.1884682

**Published:** 2026-10-09

**Authors:** Yican Hao, Xia Wei, Pingli Zhao, Hewei Qin

**Affiliations:** 1 The Second Affiliated Hospital of Henan University of Chinese Medicine, Zhengzhou, China; 2 The Second School of Clinical Medicine, Henan University of Chinese Medicine, Zhengzhou, China; 3 Zhumadian Hospital of Traditional Chinese Medicine Affiliated to Henan University of Chinese Medicine, Zhumadian, China; 4 School of Rehabilitation Medicine, Henan University of Chinese Medicine, Zhengzhou, China

**Keywords:** machine learning, post-stroke mild cognitive impairment, prediction model, scalp cluster needling therapy with prolonged needle retention, stroke rehabilitation

## Abstract

**Background:**

Cognitive recovery varies substantially among patients with post-stroke mild cognitive impairment (PSCI-MCI), whereas individualized models for predicting post-treatment cognitive improvement remain limited. This study aimed to develop and internally validate a machine learning model for predicting cognitive improvement after 1 month of scalp cluster needling therapy with prolonged needle retention (SCNT-PR) in patients with PSCI-MCI.

**Methods:**

This retrospective study included hospitalized patients diagnosed with PSCI-MCI between January 1, 2010, and January 1, 2023. Baseline data included demographics, vascular risk factors, stroke severity, imaging features, cognitive and functional assessments, emotional and sleep-related indicators, treatment-related variables, and laboratory findings. The outcome was defined as an increase of at least 2 points in the corrected Montreal Cognitive Assessment (MoCA) score after treatment compared with baseline. Least absolute shrinkage and selection operator (LASSO) regression and the Boruta algorithm were used to identify key predictors. Seven supervised machine learning algorithms were applied to develop pretreatment prediction models. Model performance was assessed in terms of discrimination, calibration, and clinical utility, and Shapley Additive Explanations (SHAP) were used for model interpretation.

**Results:**

A total of 750 patients were included and randomly assigned to a training set (*n* = 526) and a validation set (*n* = 224). Seven key predictors were identified from the intersection of LASSO regression and Boruta. Among the evaluated models, LightGBM showed the acceptable performance, with an area under the receiver operating characteristic curve of 0.759. Calibration curves and decision curve analysis indicated acceptable calibration and favorable clinical utility. SHAP analysis identified baseline corrected MoCA score, years of education, National Institutes of Health Stroke Scale total score, Barthel Index score for activities of daily living, age, frontal lobe lesion, and diabetes as the major contributors to model prediction. Sensitivity analyses demonstrated consistent predictor patterns under alternative outcome definitions and predictor configurations.

**Conclusion:**

We developed and internally validated a machine learning model based on multidimensional pretreatment clinical data to predict cognitive improvement after SCNT-PR in patients with PSCI-MCI. This model may provide preliminary support for patient stratification and individualized clinical assessment, although external validation in independent cohorts is required before clinical implementation.

## 1 Introduction

Stroke is one of the leading causes of death and disability worldwide (1). With advances in acute stroke management, survival after stroke has improved substantially; however, long-term functional impairment remains a major clinical challenge (2, 3). Among these sequelae, post-stroke cognitive impairment (PSCI) has become an important factor affecting functional recovery, quality of life, and long-term prognosis (4–6). As an important subtype of PSCI, post-stroke mild cognitive impairment (PSCI-MCI) does not meet the diagnostic threshold for dementia, but is characterized by declines in memory, attention, executive function, or information processing, and carries a risk of further progression (7, 8). Therefore, early identification and intervention for PSCI-MCI are of considerable clinical importance.

Scalp cluster needling therapy with prolonged needle retention (SCNT-PR), a traditional Chinese acupuncture-based intervention, has received increasing attention in the treatment of post-stroke dysfunction, particularly cognitive impairment. Previous studies have suggested that SCNT-PR may improve cognitive function by modulating brain activity, promoting neuroplasticity, and facilitating the reorganization of brain networks (9–11). However, in clinical practice, patients show substantial heterogeneity in their responses to the same treatment, suggesting that post-treatment cognitive improvement may be influenced by multiple baseline factors.

Although an increasing number of machine learning studies have been developed to predict the occurrence or progression of post-stroke cognitive impairment, most existing models have focused on identifying patients at risk of developing PSCI rather than predicting individualized cognitive improvement after a specific rehabilitation intervention. A recent systematic review and meta-analysis summarized 21 machine learning-based PSCI prediction studies, highlighting the rapid development of this field. However, models specifically designed to predict cognitive improvement following scalp cluster needling therapy in patients with PSCI-MCI remain scarce (12). Traditional statistical approaches have certain limitations in handling high-dimensional data, complex nonlinear relationships, and interactions among variables, whereas machine learning methods offer clear advantages in multivariable integration, feature selection, and individualized risk prediction (13, 14). In clinical practice, one of the major challenges is that patients with PSCI-MCI do not respond uniformly to the same treatment regimen. At present, clinicians lack a practical pretreatment tool to identify which patients are more likely to achieve meaningful cognitive improvement after SCNT-PR. This uncertainty may limit individualized treatment planning, reduce the efficiency of rehabilitation resource allocation, and delay optimization of therapeutic strategies. Therefore, developing a prediction model based on routinely available baseline clinical data may help address this unmet clinical need by supporting early prediction of cognitive improvement and individualized clinical assessment and more precise decision-making. Accordingly, this study retrospectively collected clinical data from patients with PSCI-MCI, identified key baseline predictors associated with cognitive improvement after SCNT-PR through feature selection, and developed and internally validated pretreatment prediction models using multiple machine learning algorithms, with the aim of providing a reference for precision intervention in clinical practice.

## 2 Materials and methods

### 2.1 Study design

This was a single-center retrospective study. Clinical data from patients with PSCI-MCI who were hospitalized at the Second Affiliated Hospital of Henan University of Chinese Medicine between January 1, 2010 and January 1, 2023 were retrospectively collected. Cognitive improvement after 1 month of SCNT-PR was analyzed, and a prediction model for cognitive improvement was developed based on pretreatment clinical data. This study was conducted in accordance with the principles of the Declaration of Helsinki. Because of the retrospective design, all patient information was anonymized before analysis. The study protocol was approved by the Ethics Committee of the Second Affiliated Hospital of Henan University of Chinese Medicine.

### 2.2 Patients and data

Patients hospitalized at the Second Affiliated Hospital of Henan University of Chinese Medicine between January 1, 2010 and January 1, 2023 who were diagnosed with PSCI-MCI at admission or during hospitalization were eligible for inclusion. The diagnosis of PSCI-MCI was based on the DSM-5 definition of mild neurocognitive disorder, together with the NINDS–Canadian Stroke Network vascular cognitive impairment harmonization standards and contemporary guidance on post-stroke cognitive impairment (15–18). Specifically, patients were required to have stroke-related mild cognitive decline involving memory, attention, executive function, language, or other cognitive domains; cognitive impairment below the expected level for age but not meeting the diagnostic threshold for dementia (19); preservation of basic activities of daily living; and no evidence of neurodegenerative disease, psychiatric disorder, or external factors that could otherwise explain the cognitive decline. In addition, scale-based assessment was required, including a corrected Montreal Cognitive Assessment (MoCA) score of <26 and a Mini-Mental State Examination (MMSE) score of 24–30 before treatment (20–23). The Chinese version of the Montreal Cognitive Assessment (MoCA) was used to evaluate global cognitive function. Education adjustment was applied according to the standard MoCA scoring procedure, with 1 additional point added for participants with ≤12 years of formal education. The adjusted MoCA score was used for subsequent analyses. A MoCA score <26 was used to identify mild cognitive impairment because of its higher sensitivity for detecting subtle cognitive deficits, whereas an MMSE score of 24–30 was additionally required to exclude patients with more severe cognitive impairment and to ensure that the study population represented mild cognitive impairment rather than dementia.

The exclusion criteria were as follows: (1) age <18 years; (2) a confirmed diagnosis of dementia before treatment; (3) disturbance of consciousness, severe visual or hearing impairment, or other conditions leading to unreliable cognitive assessment; (4) severe dysfunction of major organs such as the heart, liver, or kidneys, malignant tumors, or other serious diseases that could substantially affect prognosis; and (5) incomplete clinical data. If a patient had multiple hospitalizations during the study period, only the first hospitalization was included in the analysis.

A total of 69 pretreatment variables were collected and categorized into the following domains: (1) demographic characteristics, including age, sex, years of education, and occupation type; (2) stroke-related history and vascular risk factors, including hypertension, diabetes, atrial fibrillation, coronary heart disease, dyslipidemia, smoking history, alcohol history, prior stroke/transient ischemic attack, prior cognitive impairment, and post-stroke seizure history; (3) imaging and lesion characteristics, including mass effect, midline shift, intraventricular extension, multifocal lesions, frontal lobe involvement, temporal lobe involvement, parietal lobe involvement, occipital lobe involvement, thalamic involvement, basal ganglia involvement, cerebellar involvement, brainstem involvement, stroke type, lesion side, lesion size grade, Fazekas periventricular hyperintensity score (Fazekas_pvh), Fazekas deep white matter hyperintensity score (Fazekas_dwmh), Fazekas total score, and brain atrophy; (4) neurological, cognitive, and functional assessments, including premorbid modified Rankin Scale (mRS), National Institutes of Health Stroke Scale total score (NIHSS total score), NIHSS language score, NIHSS neglect score, baseline corrected MoCA score, baseline MMSE score, ADL score, prior stroke count, and disease duration (months); (5) emotional and sleep-related indicators, including Self-Rating Depression Scale (SDS) standard score, Pittsburgh Sleep Quality Index (PSQI), sleep apnea, and daytime fatigue; (6) treatment-related variables, including baseline rehabilitation, baseline use of cholinesterase inhibitors, memantine, anticoagulants, antiplatelet agents, cerebral metabolic drugs, and medication compliance; and (7) vital signs and laboratory findings, including systolic blood pressure, diastolic blood pressure, heart rate, fasting glucose, total cholesterol, low-density lipoprotein cholesterol (LDL-C), triglycerides, high-density lipoprotein cholesterol (HDL-C), serum creatinine, blood urea nitrogen, white blood cell count, hemoglobin, platelet count, prothrombin time, international normalized ratio (INR), and D-dimer.

During the study period from 2010 to 2023, cognitive assessment, neuroimaging evaluation, and SCNT-PR treatment were performed according to the routine clinical protocols of our institution. To minimize potential temporal heterogeneity, standardized institutional protocols for cognitive assessment, neuroimaging evaluation, and SCNT-PR administration were maintained throughout the study period. The same diagnostic framework, education-adjusted Chinese MoCA scoring procedure, and standardized treatment protocol were applied across the entire cohort. Although calendar-year information was not retained in the final analytical dataset and formal temporal validation could not be performed, this potential source of bias was considered during interpretation and is acknowledged as a limitation. The Chinese version of the MoCA with education adjustment was consistently used for cognitive assessment, and SCNT-PR was administered following a standardized institutional protocol regarding acupoint selection, needle retention duration, and treatment frequency. For variables with repeated measurements, the first pretreatment value was used for analysis. All clinical data were independently reviewed and extracted by two experienced physicians using a standardized data collection form. Any discrepancies were resolved by a third reviewer or by team discussion until consensus was reached.

The primary outcome of this study was cognitive improvement after 1 month of SCNT-PR. Cognitive improvement was defined as an increase of at least 2 points in the corrected MoCA score after treatment compared with baseline (24–26) (Outcome = 1); otherwise, the patient was classified as having no improvement (Outcome = 0). The threshold of a ≥2-point increase was selected based on previous studies reporting clinically meaningful MoCA changes after stroke. Considering that reported MCID estimates vary across populations, sensitivity analysis using a stricter threshold of MoCA ≥4 points was additionally performed to evaluate the robustness of the outcome definition.

### 2.3 Treatment protocol

All enrolled patients received standardized scalp cluster needling therapy with prolonged needle retention. Baihui (GV20), Yintang (GV29), Shenting (GV24), Sishencong (EX-HN1), and Fengfu (GV16) were selected as the acupuncture points. Disposable sterile acupuncture needles (Suzhou Medical Supplies Factory Co., Ltd.; registration number: Su Xie Zhu Zhun 20162270970; 0.30 mm × 40 mm) were used. After routine disinfection, each acupoint was punctured horizontally to a depth of approximately 1.5–2.5 cm using a balanced reinforcing-reducing method. After obtaining deqi, each needle was rotated for 1 min at a frequency of 180–200 times/min. The needles were retained for 6 h. Treatment was administered once daily for 6 consecutive days, followed by 1 day of rest, for a total treatment duration of 1 month. All procedures were performed by qualified supervising therapists.

### 2.4 Feature selection and model development

The overall workflow of this study is shown in Figure 1. Patients were randomly divided into a training set and a validation set at a ratio of 7:3. The training set was used for feature selection and model training, whereas the validation set was used for performance evaluation.

**Figure 1:**
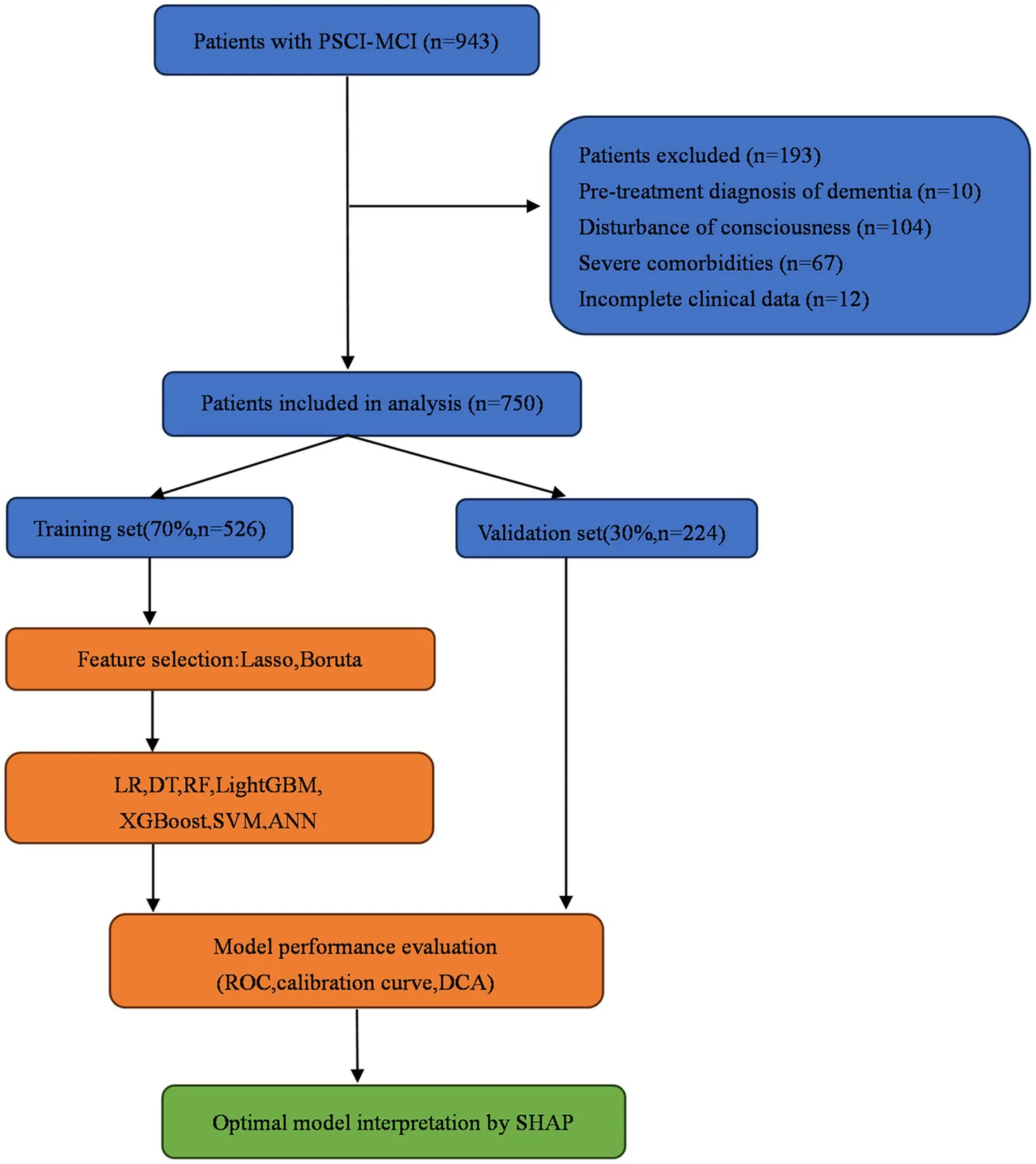
Study flow chart of patient selection, feature selection, model development, model evaluation, and SHAP-based interpretation. PSCI-MCI, post-stroke mild cognitive impairment; SCNT-PR, scalp cluster needling therapy with prolonged needle retention; LASSO, least absolute shrinkage and selection operator; LR, logistic regression; RF, random forest; SVM, support vector machine; ANN, artificial neural network; DT, decision tree; XGBoost, extreme gradient boosting; ROC, receiver operating characteristic; AUC, area under the receiver operating characteristic curve; DCA, decision curve analysis; SHAP, Shapley Additive Explanations.

Two independent methods were used in the training set to identify potential predictors: least absolute shrinkage and selection operator (LASSO) regression and the Boruta algorithm. LASSO regression performs feature selection by penalizing regression coefficients, thereby reducing multicollinearity and lowering the risk of overfitting; variables with non-zero coefficients were considered candidate predictors. The Boruta algorithm is a random forest-based feature selection method that iteratively removes variables whose importance does not significantly exceed that of shadow features generated from random noise. The intersection of variables selected by LASSO regression and the Boruta algorithm was retained as the final feature set for model development.

Based on the selected key predictors, supervised machine learning models were developed to predict cognitive improvement after 1 month of SCNT-PR in patients with PSCI-MCI. Hyperparameters were tuned using grid search combined with five-fold cross-validation to improve model stability and generalizability. Seven supervised machine learning algorithms were constructed, including logistic regression (LR), random forest (RF), support vector machine (SVM), artificial neural network (ANN), decision tree (DT), Light Gradient Boosting Machine (LightGBM), and extreme gradient boosting (XGBoost).

### 2.5 Model evaluation

Model performance was evaluated in terms of discrimination, calibration, and clinical utility to identify the optimal model. Discrimination was assessed using the receiver operating characteristic (ROC) curve and the area under the curve (AUC); a larger AUC indicates a better ability of the model to distinguish between outcome classes. Calibration was assessed using calibration curves, with curves closer to the ideal diagonal line indicating better agreement between predicted probabilities and observed outcomes. Clinical utility was evaluated using decision curve analysis (DCA), which measures the net benefit of the model across a range of threshold probabilities. Given the imbalance between improvement and no-improvement groups, precision-recall area under the curve (PR-AUC) was additionally calculated as an imbalance-aware metric to complement ROC-AUC evaluation.

### 2.6 Model interpretation

To improve model interpretability, Shapley Additive Explanations (SHAP) were applied to the optimal model. SHAP values were used to quantify the marginal contribution of each variable to the model output, thereby assessing feature importance and effect direction. SHAP plots were generated to visualize the influence of key features on the prediction of cognitive improvement.

### 2.7 Statistical analysis

Because this was a retrospective study based on available eligible cases, no formal prospective sample size calculation was performed before data collection. The adequacy of the sample size for model development was assessed using the events-per-variable principle. In this study, the less frequent outcome category was the no-improvement group, which included 212 patients. Seven predictors were retained in the final model; therefore, the events-per-variable value was 30.3, exceeding the commonly used minimum requirement of 10 events per predictor. Thus, the available sample size was considered acceptable for developing the final prediction model. Patient-level calendar year of hospitalization and therapist-specific identifiers were not collected in the analytical dataset. Therefore, temporal-period and therapist-level sensitivity analyses were not performed.

Continuous variables with a normal distribution are presented as mean ± standard deviation (SD), whereas non-normally distributed continuous variables are presented as median (interquartile range [IQR]). Categorical variables are presented as frequencies and percentages. In variable coding, binary variables were encoded as 0/1, where “1” indicated “yes” and “0” indicated “no”; for sex, “1” indicated male and “0” indicated female. Nominal multicategorical variables included stroke type (ischemic = 1, hemorrhagic = 2, other = 3), lesion side (left = 0, right = 1, bilateral = 2), and occupation type (physical labor = 1, mental labor = 2, mixed = 3). Ordinal multicategorical variables were encoded according to increasing category levels.

Between-group comparisons were performed using the independent-samples *t*-test or Mann–Whitney *U* test for continuous variables, and the chi-square test or Fisher’s exact test for categorical variables, as appropriate. A two-sided *p*-value <0.05 was considered statistically significant. The hyperparameter configurations of all machine learning models were extracted from the trained model objects and are provided in Supplementary Table 2.

Only patients with complete clinical, laboratory, and imaging information required for model development were included in the final analysis. Missing data patterns among candidate predictors were assessed before model development. Among the 943 initially screened patients, missing values were mainly observed in clinical assessment, laboratory, and imaging variables and represented the main reason for exclusion from the final analytical cohort. After applying predefined eligibility and data completeness criteria, 750 patients with complete predictor information were included in the final analysis. Missing data imputation was not performed because imputation-derived values may introduce additional uncertainty into individual-level predictions in this retrospective clinical prediction setting. A detailed summary of predictor-level missingness in the initial screening cohort is provided in Supplementary Table 5. To preserve the originality and interpretability of the data, continuous variables were not standardized for descriptive statistics or conventional statistical comparisons. Statistical analyses were performed in R. LASSO regression and Boruta analyses were conducted using the R packages “glmnet” and “Boruta,” respectively. Machine learning model development, performance evaluation, and SHAP-based interpretation were performed using Python (version 3.12). The Python environment included scikit-learn (version 1.9.0), xgboost (version 3.4.1), lightgbm (version 4.7.0), SHAP (version 0.52.0), pandas (version 3.0.5), numpy (version 2.5.2), scipy (version 1.18.1), matplotlib (version 3.11.1), and seaborn (version 0.13.2). The analysis scripts were developed and executed using Visual Studio Code. This study was reported in accordance with the TRIPOD+AI recommendations for clinical prediction model studies (Supplementary Table 3).

## 3 Results

### 3.1 Baseline characteristics

The baseline characteristics of the study population are presented in Table 1. Among the 943 initially screened patients, 750 patients were included in the final analysis after applying predefined eligibility criteria and data completeness requirements. The remaining patients were excluded because of incomplete required clinical information or failure to meet the predefined inclusion criteria. Among the 750 included patients, 212 were assigned to the no-improvement group and 538 to the improvement group. Compared with the no-improvement group, the improvement group had a higher proportion of frontal lobe lesions and baseline rehabilitation, but lower proportions of diabetes and brain atrophy. In addition, patients in the improvement group were older, had more years of education, higher ADL scores, lower baseline corrected MoCA scores, and a greater number of prior strokes. NIHSS total score also differed significantly between the two groups. No statistically significant differences were observed in the remaining baseline variables.

**Table 1:** Baseline characteristics of patients in the no-improvement and improvement groups.

Variable	Overall (*n* = 750)	No improvement (*n* = 212)	Improvement (*n* = 538)	*p*
Male (%)	378 (50.40)	105 (49.53)	273 (50.74)	0.827
Mass effect (%)	75 (10.00)	18 (8.49)	57 (10.59)	0.466
Midline shift (%)	40 (5.33)	16 (7.55)	24 (4.46)	0.13
Intraventricular extension (%)	62 (8.27)	20 (9.43)	42 (7.81)	0.561
Lesion multifocal (%)	213 (28.40)	65 (30.66)	148 (27.51)	0.44
Lesion frontal (%)	277 (36.93)	50 (23.58)	227 (42.19)	<0.001
Lesion temporal (%)	260 (34.67)	65 (30.66)	195 (36.25)	0.173
Lesion parietal (%)	187 (24.93)	60 (28.30)	127 (23.61)	0.213
Lesion occipital (%)	109 (14.53)	30 (14.15)	79 (14.68)	0.943
Lesion thalamus (%)	150 (20.00)	42 (19.81)	108 (20.07)	1
Lesion basalganglia (%)	146 (19.47)	39 (18.40)	107 (19.89)	0.717
Lesion cerebellum (%)	64 (8.53)	14 (6.60)	50 (9.29)	0.297
Lesion brainstem (%)	24 (3.20)	9 (4.25)	15 (2.79)	0.429
Hypertension (%)	448 (59.73)	117 (55.19)	331 (61.52)	0.131
Diabetes (%)	197 (26.27)	70 (33.02)	127 (23.61)	0.011
Atrial fibrillation (%)	130 (17.33)	33 (15.57)	97 (18.03)	0.487
Coronary heart disease (%)	139 (18.53)	43 (20.28)	96 (17.84)	0.503
Dyslipidemia (%)	286 (38.13)	75 (35.38)	211 (39.22)	0.372
Smoking history (%)	226 (30.13)	62 (29.25)	164 (30.48)	0.807
Alcohol history (%)	154 (20.53)	41 (19.34)	113 (21.00)	0.684
Prior stroke/TIA (%)	129 (17.20)	38 (17.92)	91 (16.91)	0.824
Prior cognitive impairment (%)	99 (13.20)	27 (12.74)	72 (13.38)	0.908
Poststroke seizure history (%)	43 (5.73)	13 (6.13)	30 (5.58)	0.904
Baseline rehab (%)	453 (60.40)	113 (53.30)	340 (63.20)	0.016
Chei use baseline (%)	250 (33.33)	80 (37.74)	170 (31.60)	0.129
Memantine use baseline (%)	91 (12.13)	29 (13.68)	62 (11.52)	0.49
Anticoagulant use baseline (%)	137 (18.27)	36 (16.98)	101 (18.77)	0.64
Antiplatelet use baseline (%)	512 (68.27)	137 (64.62)	375 (69.70)	0.208
Cerebral metabolic drug use baseline (%)	187 (24.93)	49 (23.11)	138 (25.65)	0.529
Brain atrophy (%)	253 (33.73)	84 (39.62)	169 (31.41)	0.04
Sleep apnea (%)	103 (13.73)	27 (12.74)	76 (14.13)	0.704
Daytime fatigue (%)	194 (25.87)	54 (25.47)	140 (26.02)	0.95
Medication compliance (%)	609 (81.20)	175 (82.55)	434 (80.67)	0.625
Stroke type (%)				0.372
Ischemic	603 (80.40)	171 (80.66)	432 (80.30)	
Hemorrhagic	110 (14.67)	34 (16.04)	76 (14.13)	
Other	37 (4.93)	7 (3.30)	30 (5.58)	
Lesion side (%)				0.99
Left	312 (41.60)	89 (41.98)	223 (41.45)	
Right	302 (40.27)	85 (40.09)	217 (40.33)	
Bilateral	136 (18.13)	38 (17.92)	98 (18.22)	
Lesion size grade (%)				0.17
Small	236 (31.47)	67 (31.60)	169 (31.41)	
Medium	296 (39.47)	93 (43.87)	203 (37.73)	
Big	218 (29.07)	52 (24.53)	166 (30.86)	
Fazekas pvh (%)				0.354
None	310 (41.33)	89 (41.98)	221 (41.08)	
Mild	224 (29.87)	62 (29.25)	162 (30.11)	
Moderate	137 (18.27)	33 (15.57)	104 (19.33)	
Severe	79 (10.53)	28 (13.21)	51 (9.48)	
Fazekas dwmh (%)				0.646
None	327 (43.60)	93 (43.87)	234 (43.49)	
Mild	226 (30.13)	66 (31.13)	160 (29.74)	
Moderate	132 (17.60)	39 (18.40)	93 (17.29)	
Severe	65 (8.67)	14 (6.60)	51 (9.48)	
Fazekas total (%)				0.538
None	303 (40.40)	81 (38.21)	222 (41.26)	
Mild	239 (31.87)	72 (33.96)	167 (31.04)	
Moderate	153 (20.40)	40 (18.87)	113 (21.00)	
Severe	55 (7.33)	19 (8.96)	36 (6.69)	
Premorbid mrs (%)				0.34
None	381 (50.80)	112 (52.83)	269 (50.00)	
Mild symptoms	226 (30.13)	56 (26.42)	170 (31.60)	
Mild disability	72 (9.60)	23 (10.85)	49 (9.11)	
Moderate disability	39 (5.20)	14 (6.60)	25 (4.65)	
Moderate to severe disability	17 (2.27)	2 (0.94)	15 (2.79)	
Severe disability	15 (2.00)	5 (2.36)	10 (1.86)	
Occupation type (%)				0.235
Physical labor	302 (40.27)	77 (36.32)	225 (41.82)	
Mental labor	210 (28.00)	68 (32.08)	142 (26.39)	
Mixture	238 (31.73)	67 (31.60)	171 (31.78)	
NIHSS language (%)				0.326
Normal	440 (58.67)	123 (58.02)	317 (58.92)	
Mild-to-moderate	193 (25.73)	62 (29.25)	131 (24.35)	
Severe	72 (9.60)	15 (7.08)	57 (10.59)	
Global aphasia	45 (6.00)	12 (5.66)	33 (6.13)	
NIHSS neglect (%)				0.738
None	526 (70.13)	146 (68.87)	380 (70.63)	
Mild	146 (19.47)	45 (21.23)	101 (18.77)	
Severe	78 (10.40)	21 (9.91)	57 (10.59)	
Age (years)	61.00 [49.82, 69.47]	55.25 [47.18, 65.03]	62.95 [52.73, 71.20]	<0.001
Education years (years)	10.25 [4.82, 14.70]	8.30 [3.68, 13.43]	10.55 [5.50, 15.38]	0.001
Disease duration (months)	7.97 [3.46, 15.62]	8.40 [3.65, 16.69]	7.57 [3.36, 14.96]	0.537
NIHSS total score (points)	2.00 [1.00, 3.00]	2.00 [1.00, 3.00]	2.00 [1.00, 3.00]	<0.001
ADL score (points)	85.90 [77.70, 93.00]	83.10 [75.97, 90.88]	86.95 [78.65, 93.38]	0.001
MoCA pre (points)	21.40 [19.80, 23.20]	22.60 [20.98, 23.90]	21.00 [19.50, 22.70]	<0.001
MMSE pre (points)	27.10 [25.60, 28.60]	26.70 [25.50, 28.30]	27.15 [25.60, 28.70]	0.194
SDS standard score (points)	49.65 [42.50, 56.20]	50.90 [42.90, 56.42]	49.50 [42.32, 56.00]	0.295
PSQI score (points)	5.00 [3.00, 6.00]	5.00 [3.00, 6.00]	5.00 [3.25, 6.00]	0.726
SBP (mmHg)	129.70 [119.23, 139.50]	129.25 [117.58, 139.57]	130.20 [120.05, 139.48]	0.541
DBP (mmHg)	79.55 [72.90, 86.70]	79.70 [73.68, 86.75]	79.50 [72.43, 86.70]	0.438
Heart rate (bpm)	74.95 [68.30, 82.18]	74.40 [67.97, 80.53]	75.25 [68.43, 82.40]	0.315
Fasting glucose (mg/dL)	94.30 [84.50, 104.50]	94.15 [85.50, 104.32]	94.30 [84.32, 104.68]	0.924
Total cholesterol (mg/dL)	188.75 [170.83, 206.95]	186.05 [170.15, 205.43]	189.55 [171.12, 207.30]	0.25
LDL-C (mg/dL)	110.20 [91.95, 128.98]	112.05 [90.50, 129.25]	109.50 [92.60, 128.60]	0.939
Triglycerides (mg/dL)	92.15 [69.25, 129.83]	90.85 [67.15, 121.73]	93.05 [69.62, 131.55]	0.425
HDL-C (mg/dL)	51.00 [43.82, 57.20]	50.90 [43.38, 57.60]	51.00 [43.92, 57.18]	0.955
Creatinine (mg/dL)	0.90 [0.75, 1.03]	0.88 [0.74, 1.06]	0.91 [0.75, 1.02]	0.939
BUN (mg/dL)	14.00 [11.20, 16.80]	13.75 [10.67, 16.20]	14.10 [11.40, 17.00]	0.097
WBC (×10^9^/L)	7.02 [6.04, 8.11]	6.94 [6.08, 7.89]	7.06 [6.00, 8.30]	0.311
Hemoglobin (g/dL)	14.00 [13.00, 15.00]	14.05 [12.90, 15.10]	13.90 [13.10, 14.90]	0.678
Platelets (×10^9^/L)	253.95 [217.62, 287.60]	250.70 [217.75, 283.15]	254.85 [217.62, 289.03]	0.409
PT (s)	11.99 [11.32, 12.60]	12.08 [11.38, 12.67]	11.97 [11.31, 12.59]	0.159
INR	1.10 (0.10)	1.10 (0.10)	1.10 (0.10)	0.776
D-dimer (ng/mL)	73.05 [32.05, 142.43]	76.05 [36.35, 143.27]	72.15 [31.72, 140.82]	0.691
Prior stroke count	0.00 [0.00, 1.00]	0.00 [0.00, 1.00]	0.00 [0.00, 1.00]	0.008

Data are presented as median [interquartile range (IQR)], mean (standard deviation, SD), or *n* (%), as appropriate. *p*-values were calculated using the Chi-square test or Fisher’s exact test for categorical variables and the independent-samples *t*-test or Mann–Whitney *U* test for continuous variables, as appropriate. PSCI-MCI, post-stroke mild cognitive impairment; SCNT-PR, scalp cluster needling therapy with prolonged needle retention; MoCA, Montreal Cognitive Assessment; MMSE, mini-mental state examination; NIHSS, National Institutes of Health Stroke Scale; ADL, Barthel index score for activities of daily living; SDS, Self-Rating Depression Scale; PSQI, Pittsburgh Sleep Quality Index; SBP, systolic blood pressure; DBP, diastolic blood pressure; LDL-C, low-density lipoprotein cholesterol; HDL-C, high-density lipoprotein cholesterol; BUN, blood urea nitrogen; WBC, white blood cell count; PT, prothrombin time; INR, international normalized ratio.

### 3.2 Model development

Among all baseline features, LASSO regression identified 8 candidate predictors with non-zero coefficients based on cross-validation (Figures 2A,B). Meanwhile, the Boruta algorithm identified 7 potentially important predictors according to feature importance (Figures 2C,D). The intersection of the two feature selection methods (Figure 2E) yielded 7 shared predictors, including frontal lobe lesion, diabetes, age, years of education, NIHSS total score, ADL score, and baseline corrected MoCA score.

**Figure 2:**
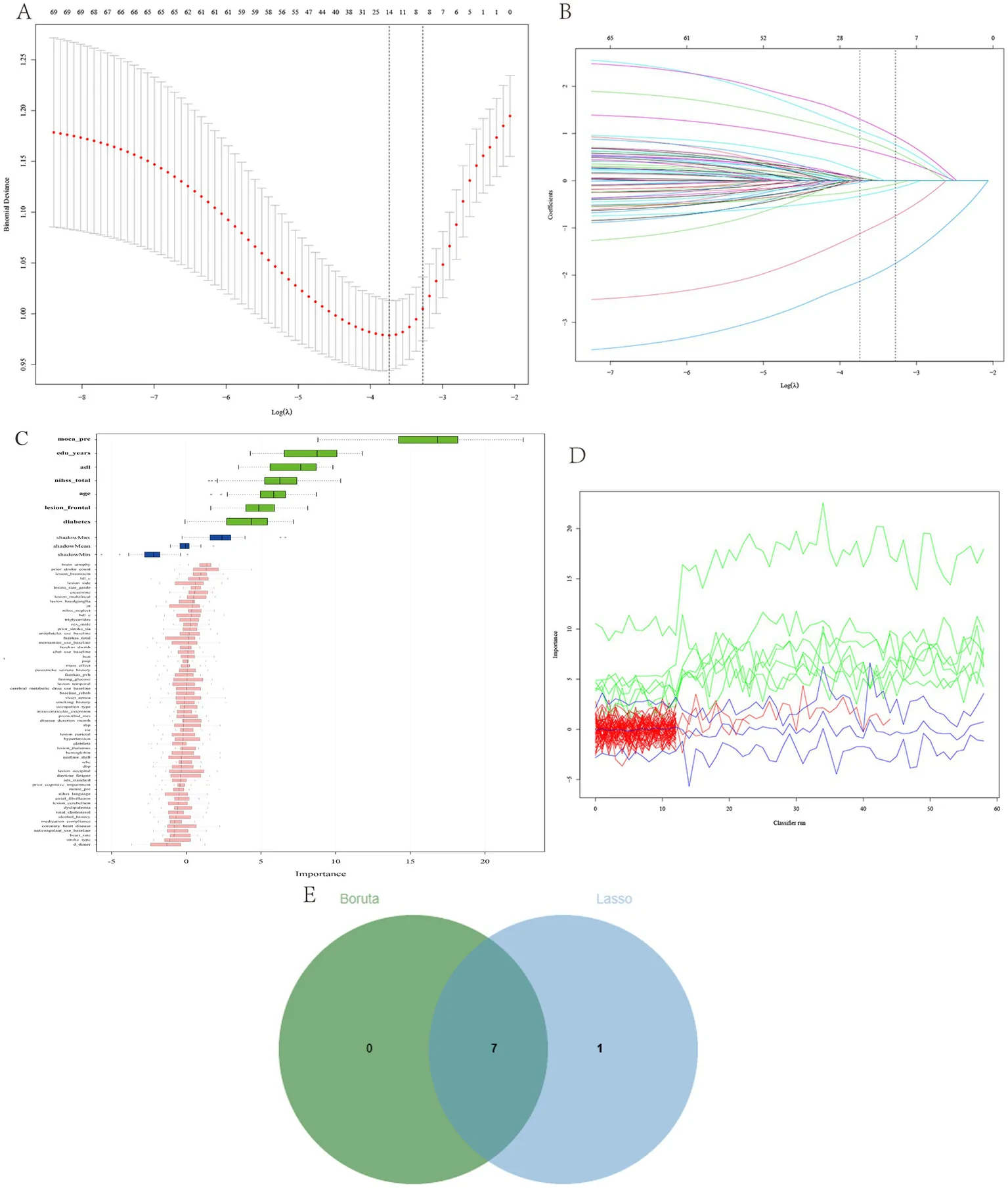
Feature selection using LASSO regression and the Boruta algorithm. (**A)** Ten-fold cross-validation curve for selection of the optimal tuning parameter (*λ*) in the LASSO regression model. The two vertical dashed lines indicate the optimal *λ* values under the minimum criterion and the 1 − standard-error (1 − SE) criterion, respectively. **(B)** LASSO coefficient profiles of all baseline features. Each colored line represents the coefficient trajectory of an individual variable as the value of log (*λ*) changes. **(C)** Variable importance plot generated by the Boruta algorithm. Green variables represent confirmed important features, blue variables indicate shadow features generated from randomized variables, and red variables represent rejected features. **(D)** Iterative changes in feature importance during the Boruta procedure. **(E)** Venn diagram showing the overlap between variables selected by LASSO regression and the Boruta algorithm. A total of 7 shared predictors were identified. LASSO, least absolute shrinkage and selection operator; SE, standard error.

### 3.3 Model performance, discrimination, calibration, and clinical utility

The predictive performance of the seven machine learning models in the validation set is summarized in Table 2 and Figure 3. Overall, all models showed a certain ability to predict cognitive improvement. Among them, the Light Gradient Boosting Machine (LightGBM) model achieved the best discriminative performance, with an AUC of 0.759 (95% CI: 0.693–0.820). The logistic regression and support vector machine (SVM) models showed comparable performance, with AUCs of 0.749 (95% CI: 0.679–0.816) and 0.748 (95% CI: 0.679–0.813), respectively. Random forest, XGBoost, and artificial neural network (ANN) models also showed good discrimination, with AUCs of 0.744, 0.744, and 0.741, respectively. In contrast, the decision tree model performed the worst, with an AUC of 0.638 (95% CI: 0.559–0.713). Because the outcome distribution was imbalanced, precision-recall area under the curve (PR-AUC) was additionally calculated as an imbalance-aware performance metric. Among the evaluated models, LightGBM achieved the highest PR-AUC, indicating that it maintained favorable precision-recall performance in the validation cohort despite the imbalanced outcome distribution. Given the imbalanced outcome distribution and the relatively limited specificity at the default threshold, threshold optimization analysis was further performed for the LightGBM model. Different probability thresholds demonstrated different trade-offs between sensitivity and specificity. A higher threshold improved specificity but reduced sensitivity, indicating that threshold selection should depend on the intended clinical application (Supplementary Table 4).

**Table 2:** Predictive performance of the seven machine learning models for cognitive improvement in the validation set.

Model	AUC	PR-AUC	95% CI Lower	95% CI Upper	Accuracy	Precision	Sensitivity	Specificity	*F*1-score	Kappa	Youden’s *J*	PPV	NPV
Logistic	0.749	0.885	0.679	0.816	0.75	0.797	0.876	0.429	0.834	0.330	0.304	0.797	0.574
Decision tree	0.638	0.805	0.559	0.713	0.714	0.734	0.944	0.127	0.826	0.091	0.071	0.734	0.471
Random forest	0.744	0.881	0.672	0.810	0.732	0.770	0.894	0.317	0.828	0.242	0.212	0.770	0.541
XGBoost	0.744	0.879	0.671	0.812	0.741	0.764	0.925	0.270	0.837	0.234	0.195	0.764	0.586
LightGBM	0.759	0.900	0.693	0.820	0.714	0.787	0.826	0.429	0.806	0.265	0.255	0.787	0.491
SVM	0.748	0.884	0.679	0.813	0.741	0.784	0.882	0.381	0.830	0.291	0.263	0.785	0.558
ANN	0.741	0.883	0.670	0.808	0.728	0.791	0.845	0.429	0.817	0.289	0.273	0.791	0.519

AUC, area under the receiver operating characteristic curve; CI, confidence interval; PPV, positive predictive value; NPV, negative predictive value. Accuracy, precision, sensitivity, specificity, *F*1-score, Cohen’s kappa, and Youden’s *J* were used to assess model performance. PR-AUC, precision-recall area under the curve; PR-AUC was calculated using the validation cohort to further evaluate model performance under the imbalanced outcome distribution.

**Figure 3:**
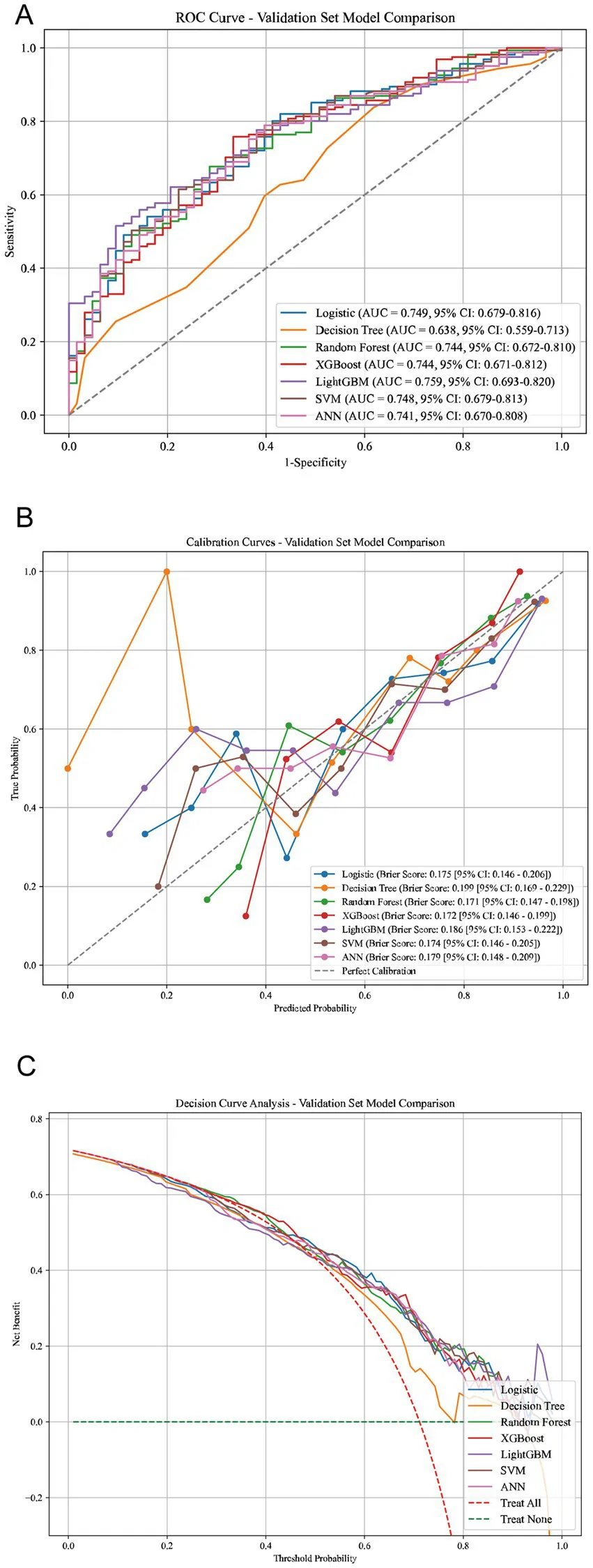
Comparison of discrimination, calibration, and clinical utility among the seven machine learning models in the validation set. **(A)** Receiver operating characteristic (ROC) curves. **(B)** Calibration curves. **(C)** Decision curve analysis (DCA). AUC, area under the receiver operating characteristic curve; CI, confidence interval.

In terms of calibration (Figure 3B), all models showed a certain degree of agreement between predicted probabilities and observed outcomes, although calibration performance varied across models. The random forest model had the lowest Brier score, at 0.171 (95% CI: 0.147–0.198), indicating relatively favorable calibration. This was followed by the XGBoost model with a Brier score of 0.172 (95% CI: 0.146–0.199), the SVM model with a Brier score of 0.174 (95% CI: 0.146–0.205), and the logistic regression model with a Brier score of 0.175 (95% CI: 0.146–0.206). The LightGBM model yielded a Brier score of 0.186 (95% CI: 0.153–0.222), indicating acceptable overall calibration. The decision tree model had the highest Brier score, at 0.199 (95% CI: 0.169–0.229), suggesting relatively inferior calibration.

Decision curve analysis (DCA) showed that, across a range of threshold probabilities, all models provided varying degrees of net benefit compared with the “treat all” and “treat none” strategies. Among them, the LightGBM model demonstrated favorable net benefit over clinically relevant threshold ranges, suggesting good potential for clinical application.

Taken together, although some models showed slightly better performance in individual classification metrics, the LightGBM model achieved the highest AUC, acceptable calibration, and favorable clinical net benefit on DCA. To further visualize classification performance, confusion matrices of the seven models in the validation set were generated (Figure 4). Overall, the models showed stronger ability to identify patients with improvement than those without improvement, which was consistent with the generally high sensitivity but relatively low specificity shown in Table 2. Although the decision tree model achieved high sensitivity, it showed the weakest ability to distinguish patients without improvement. In contrast, the LightGBM model demonstrated a better balance between positive and negative sample classification. Based on the integrated evaluation of ROC analysis, calibration characteristics, and DCA results, LightGBM was selected as the primary model for SHAP-based interpretation because it achieved the highest AUC and demonstrated a favorable overall balance between discrimination, calibration, and clinical utility among the evaluated models.

**Figure 4:**
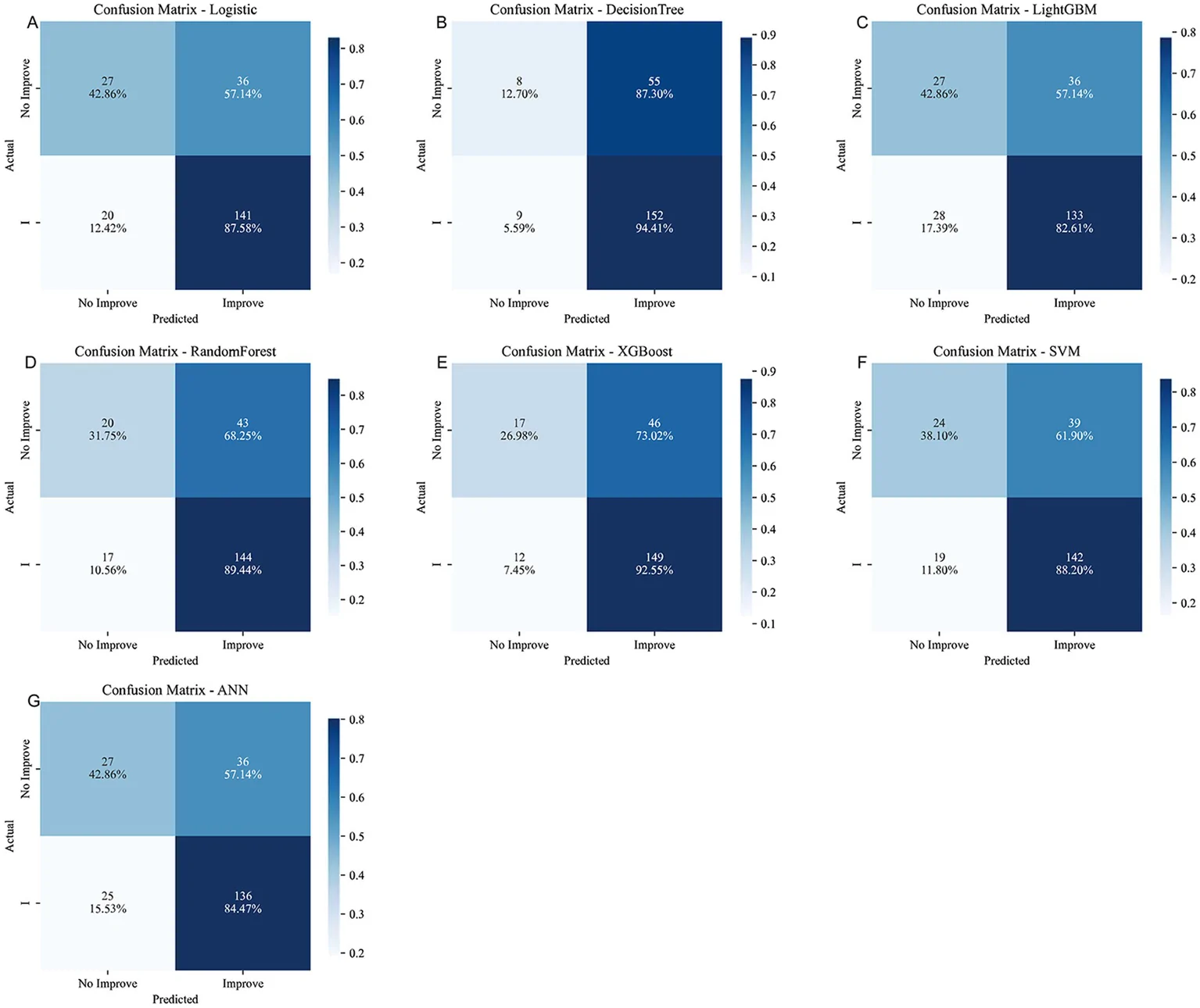
Confusion matrices of the seven machine learning models in the validation set. **(A)** Logistic regression; **(B)** decision tree; **(C)** LightGBM; **(D)** random forest; **(E)** XGBoost; **(F)** SVM; and **(G)** ANN. The confusion matrices illustrate the classification results of the seven models, including true positives, true negatives, false positives, and false negatives.

### 3.4 Model interpretation

To further improve the interpretability of the optimal model, SHAP was applied to the LightGBM model (Figure 5). The SHAP feature importance bar plot (Figure 5A) showed that the main contributors to the prediction of cognitive improvement were baseline corrected MoCA score, years of education, NIHSS total score, ADL score, age, frontal lobe lesion, and diabetes. These findings were consistent with the shared predictors identified by LASSO regression and the Boruta algorithm, indicating good stability and consistency of the final model.

**Figure 5:**
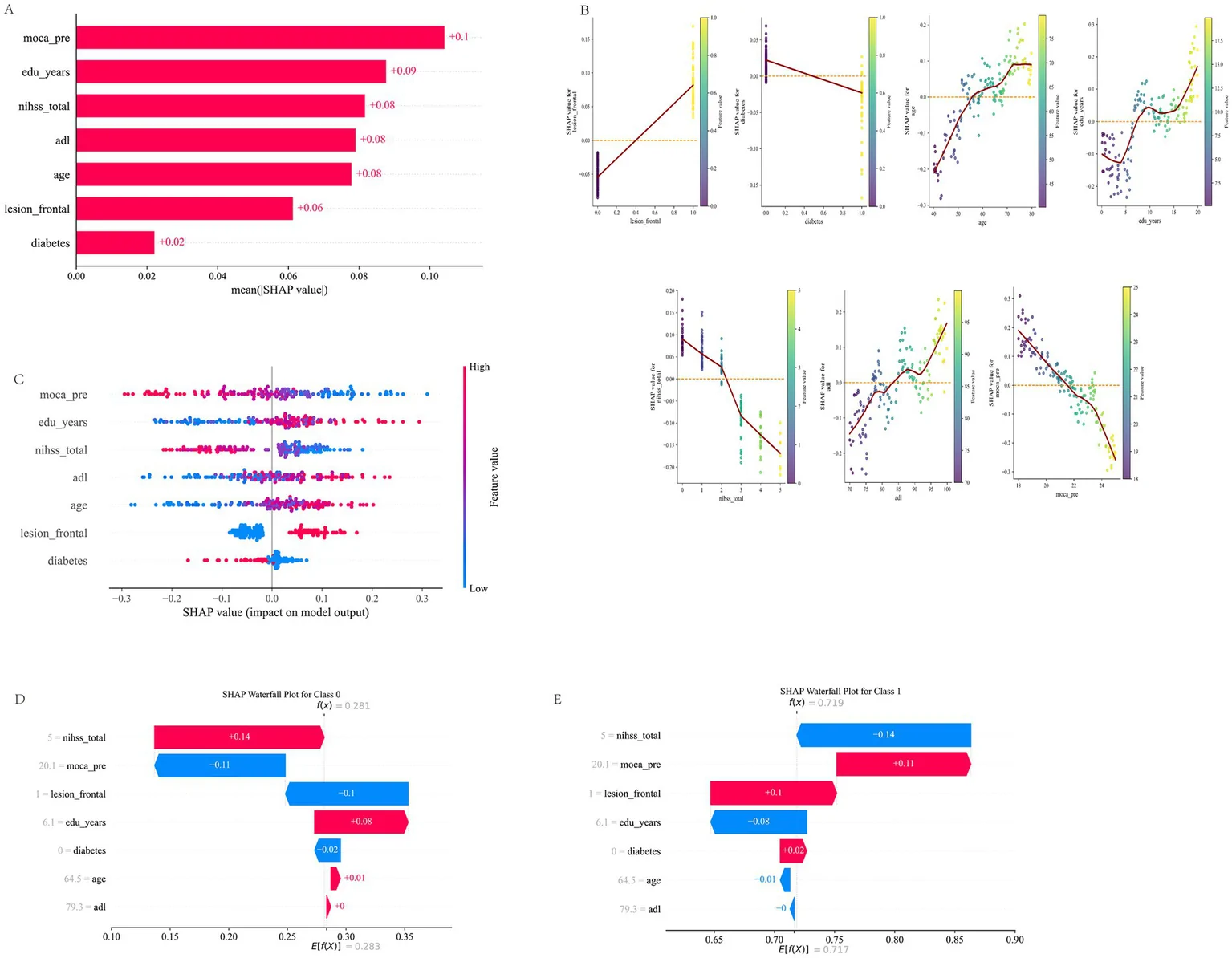
SHAP-based interpretation of the optimal LightGBM model. **(A)** SHAP feature importance bar plot showing the global importance ranking of the key predictors. **(B)** SHAP dependence plot showing the relationship between feature values and their corresponding SHAP values. **(C)** SHAP beeswarm plot showing the distribution, direction, and magnitude of feature effects across all samples. **(D,E)** SHAP waterfall plots illustrating the contributions of individual features to the model output in two representative patients. SHAP, SHapley Additive exPlanations; MoCA, Montreal Cognitive Assessment; NIHSS, National Institutes of Health Stroke Scale; ADL, activities of daily living.

The SHAP dependence plot (Figure 5B) further illustrated the relationship between feature values and the corresponding SHAP values, suggesting that the effects of key variables on model output were partly nonlinear. Meanwhile, the SHAP beeswarm plot (Figure 5C) showed the direction, magnitude, and distribution of feature effects across all samples. Overall, baseline corrected MoCA score, years of education, NIHSS total score, and ADL score had substantial influence on model prediction, while age, frontal lobe lesion, and diabetes also played important roles in model discrimination. These results indicate that baseline cognitive status, cognitive reserve, stroke severity, functional status, lesion location, and metabolic risk factors jointly influence the probability of cognitive improvement in patients with PSCI-MCI after SCNT-PR treatment.

At the individual level, the SHAP waterfall plots (Figures 5D,E) visually demonstrated the cumulative contributions of different features to the prediction in representative patients, with some variables driving the model output toward improvement and others toward no improvement. The direction and magnitude of feature contributions varied across individuals, indicating that the model can not only identify key predictors at the global level but also explain how different baseline characteristics jointly drive the final prediction at the individual level, thereby enhancing its clinical interpretability.

In the sensitivity analysis using the stricter outcome definition (MoCA ≥4 points), SHAP analysis of the XGBoost model identified baseline corrected MoCA score, ADL score, education years, frontal lobe lesion, and NIHSS total score as the major contributors to model prediction, with feature importance patterns generally consistent with the primary analysis (Supplementary Figure 1).

### 3.5 Sensitivity analyses

Two sensitivity analyses were performed to evaluate the robustness of the predictive models under alternative outcome definitions and predictor configurations. First, a stricter definition of cognitive improvement was applied by defining the outcome as an increase of ≥4 points in the corrected MoCA score after treatment. Under this alternative outcome definition, the predictive performance of the LightGBM model decreased compared with the primary analysis. The model showed reduced discrimination, with an AUC of approximately 0.66, accompanied by poorer calibration performance, suggesting decreased predictive consistency under the stricter outcome threshold (Figures 6A–C). Second, to evaluate whether the predictive performance was overly dependent on baseline cognitive status, baseline corrected MoCA score was removed from the predictor set while retaining the original outcome definition (MoCA ≥2 points). The performance of the models showed only limited changes after excluding baseline MoCA, suggesting that the predictive ability was not solely driven by baseline cognitive impairment severity (Figures 6D–F). In addition, multivariable logistic regression analysis using MoCA ≥4 points as the sensitivity outcome showed that baseline corrected MoCA score, years of education, ADL score, NIHSS total score, and frontal lobe lesion remained significant predictors, with directions consistent with the primary analysis (Supplementary Table 1).

**Figure 6:**
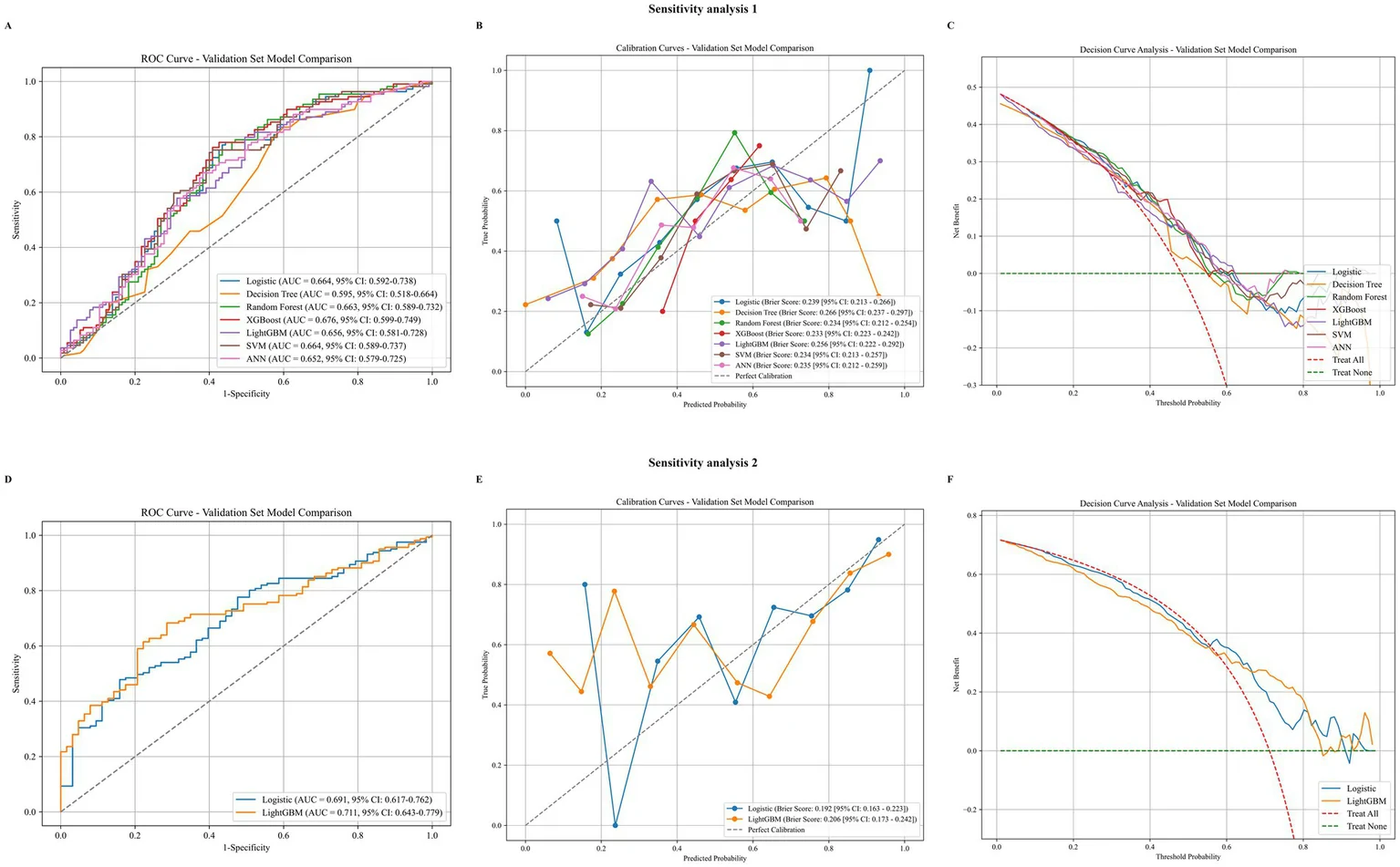
Sensitivity analyses of predictive model performance. **(A–C)** Model performance in the validation cohort using a stricter definition of cognitive improvement (MoCA ≥4 points). ROC curves **(A)**, calibration curves **(B)**, and DCA **(C)** are presented. **(D–F)** Model performance after excluding baseline MoCA from the predictor set while retaining the original outcome definition (MoCA ≥2 points). ROC curves **(D)**, calibration curves **(E)**, and DCA **(F)** are presented.

## 4 Discussion

In this study, we developed and internally validated a machine learning model based on multidimensional pretreatment clinical data to predict cognitive improvement after 1 month of SCNT-PR in patients with PSCI-MCI. The results showed that the LightGBM model achieved the best discriminative performance in the validation set, with acceptable calibration and favorable clinical net benefit. Through LASSO regression, the Boruta algorithm, and SHAP-based interpretation, we ultimately identified seven stable and clinically interpretable key predictors, namely baseline corrected MoCA score, years of education, NIHSS total score, ADL score, age, frontal lobe lesion, and diabetes. These findings suggest that cognitive improvement after SCNT-PR is associated with the combined contribution of baseline cognitive status, cognitive reserve, stroke severity, functional status, lesion characteristics, and vascular-metabolic factors. Although the identified predictors were routinely available clinical variables that could also be incorporated into conventional regression models, the objective of applying machine learning in this study was not to replace traditional statistical approaches or identify novel predictors. Instead, machine learning was used to evaluate whether nonlinear modeling strategies could capture complex relationships and potential interactions among multidimensional clinical features. Notably, the performance advantage of LightGBM over logistic regression was modest, suggesting that the added value of machine learning in this context may mainly lie in flexible integration and interpretation of multiple clinical characteristics rather than a substantial improvement in discrimination alone. Therefore, the current model should be considered a complementary prediction framework rather than a universally superior alternative to conventional regression models.

Although the LightGBM model demonstrated acceptable predictive performance, its discrimination was lower than that reported in some previous machine learning models for PSCI prediction. A systematic review and meta-analysis of 21 PSCI prediction studies reported pooled c-index values of approximately 0.82 in both training and validation cohorts (12). In addition, Lee et al. developed machine learning models for predicting PSCI after acute ischemic stroke and reported an AUC of 0.792 for the extreme gradient boosting model (27). However, these previous studies mainly focused on predicting the occurrence of post-stroke cognitive impairment rather than cognitive improvement after intervention. Compared with disease occurrence prediction, prediction of treatment-associated cognitive improvement is inherently more challenging because it may be affected by treatment response heterogeneity, spontaneous neurological recovery, repeated cognitive assessment effects, concurrent rehabilitation, and other patient-specific factors. Therefore, direct comparison of predictive performance across different clinical prediction objectives should be interpreted cautiously. By integrating routinely available baseline clinical data, the present model may provide additional information for patient stratification and individualized clinical assessment.

Although the model demonstrated acceptable discrimination, its relatively limited specificity and negative predictive value indicate that it should be interpreted as an auxiliary decision-support tool rather than a standalone instrument for excluding patients unlikely to improve. In addition, because the improvement group represented the majority of patients, class imbalance may have influenced the classification characteristics of the models. Therefore, PR-AUC was additionally evaluated, and the LightGBM model maintained the highest PR-AUC among the tested algorithms, suggesting relatively stable predictive performance beyond ROC-based assessment. Nevertheless, further studies with larger and more balanced cohorts are required to optimize decision thresholds and improve discrimination of patients less likely to improve. Threshold optimization analysis further demonstrated that alternative probability cutoffs could improve specificity at the expense of sensitivity. Therefore, the default threshold should not be considered a fixed clinical cutoff, and future studies should determine optimal thresholds according to specific clinical objectives and external validation results.

Another important consideration relates to the potential influence of baseline cognitive status on model prediction. Because the outcome was defined as change from baseline MoCA, the contribution of baseline cognitive status may partly reflect mathematical coupling and regression-to-the-mean effects. Therefore, the predictive role of baseline MoCA should be interpreted cautiously. The sensitivity analysis excluding baseline MoCA demonstrated that model performance remained relatively stable, suggesting that the predictive ability was not solely dependent on baseline cognitive status.

Notably, SHAP analysis not only revealed the importance of these predictors but also suggested that they influenced model output with different directions and magnitudes. Overall, these variables showed different patterns of contribution to the LightGBM model output. Higher education years and ADL scores were generally associated with positive SHAP contributions, whereas diabetes showed relatively negative contributions. In contrast, frontal lobe lesion and older age showed positive contributions to model prediction in this cohort. These findings indicate that the direction of individual feature contributions should be interpreted based on the observed model patterns rather than assumed biological expectations. Baseline corrected MoCA score and NIHSS total score also had substantial effects on model output, although their directions should be interpreted in the context of baseline impairment severity and potential room for improvement. It should be emphasized that SHAP reflects the marginal contribution of variables to model prediction rather than a simple positive or negative association in the traditional linear sense. Therefore, the interpretation of variable directionality in this study is based on overall trends rather than absolute linear relationships. These SHAP-based interpretations should be considered as model-derived associations rather than causal effects.

First, the baseline corrected MoCA score was one of the most important predictors identified in this study. As a widely used screening tool for post-stroke cognitive impairment, the MoCA comprehensively reflects baseline status across multiple cognitive domains, including attention, executive function, memory, language, and visuospatial ability (28). Its inclusion in the final model indicates that pretreatment cognitive status itself contains important prognostic information. According to the SHAP results, the corrected MoCA score had a substantial influence on model output, suggesting that it reflects not only the severity of cognitive impairment but also, indirectly, the potential for subsequent improvement (29). In general, a lower baseline corrected MoCA score may indicate more severe cognitive impairment, but in some patients it may also imply greater room for measurable improvement (24–26). Therefore, this variable may be better understood as a composite indicator reflecting both baseline cognitive status and improvement potential, rather than a simple one-directional predictor. Clinically, this finding highlights that standardized baseline cognitive assessment is important not only for diagnosing PSCI-MCI but also for predicting treatment response and stratifying risk.

Second, years of education served as an important indicator of cognitive reserve. Previous studies have consistently suggested that individuals with a higher educational level tend to have greater cognitive reserve and stronger compensatory capacity, which may confer better cognitive recovery potential after stroke-related brain injury (30–32). In the present study, years of education was stably retained in the final model, and SHAP results suggested that higher educational attainment generally tended to increase the predicted probability of cognitive improvement. This finding indicates that treatment outcome in PSCI-MCI is influenced not only by structural brain injury but also by the long-term accumulation of cognitive reserve. It also suggests that years of education should be considered more than a simple demographic characteristic, but rather an important background variable in cognitive prognostic assessment (33). Patients with fewer years of education may require closer monitoring and more targeted rehabilitation support during treatment.

Third, the NIHSS total score reflects the overall severity of neurological deficits after stroke and is a well-established measure of stroke severity (34, 35). Although the study population consisted of patients with PSCI-MCI and the overall NIHSS scores were relatively low, this variable still entered the final model, suggesting that even mild differences in neurological deficit may affect subsequent cognitive improvement (36). A higher NIHSS total score usually implies involvement of broader or more critical brain networks and may therefore compromise cognitive recovery capacity (37). The SHAP results also indicated that the NIHSS total score contributed substantially to model output, with higher NIHSS levels generally tending to be unfavorable for cognitive improvement. This finding further supports the close relationship between stroke severity and cognitive outcome and suggests that the prognostic value of NIHSS should not be overlooked even in relatively mild PSCI-MCI populations.

Fourth, ADL score was another important predictor in the final model. As a commonly used measure of activities of daily living, the Barthel Index reflects functional independence and overall recovery status after stroke (38). Although basic daily functioning is usually preserved in patients with PSCI-MCI, subtle functional decline may still be present (39). The inclusion of ADL score in the final model suggests that even mild differences in functional status may, to some extent, reflect the degree of cognitive impairment, brain injury burden, and rehabilitation potential. SHAP results further showed that higher ADL scores generally tended to increase the predicted probability of cognitive improvement (40, 41). This finding suggests that better baseline functional status may indicate greater treatment adherence, stronger recovery potential, and more preserved functional reserve. Therefore, incorporating functional status into the prediction of cognitive outcome appears to be necessary and clinically meaningful.

Fifth, age was identified as another key predictor. Increasing age is generally accompanied by reduced brain reserve, diminished neuroplasticity, greater white matter injury, more pronounced brain atrophy, and a higher burden of chronic vascular risk factors, all of which may jointly impair post-stroke cognitive recovery (42–44). The inclusion of age in the final model indicates that it remains a fundamental factor in predicting cognitive improvement in PSCI-MCI. SHAP analysis suggested that older age contributed positively to the predicted probability of cognitive improvement in this cohort. Although advanced age is traditionally considered to be associated with reduced cognitive reserve and impaired neuroplasticity, the observed direction in this model may reflect the complex interactions among baseline characteristics, disease stage, rehabilitation exposure, and treatment response patterns within the studied population. However, the role of age in this study should be interpreted as reflecting an overall risk background rather than a purely deterministic factor. Clinically, older patients are not necessarily unable to improve, but they often require more cautious prognostic evaluation and more individualized intervention strategies.

Sixth, the frontal lobe plays a central role in executive function, attentional control, planning, behavioral regulation, and working memory, all of which are cognitive domains commonly impaired in PSCI-MCI (45–48). Its retention in the final model suggests that lesion location has important value in predicting post-stroke cognitive outcome. Compared with broader imaging descriptors such as stroke type or lesion size alone, frontal lobe involvement may more directly reflect injury to brain regions critical for cognition. SHAP analysis further suggested that frontal lobe lesion contributed positively to the predicted probability of cognitive improvement in this cohort. Although frontal lobe involvement is traditionally considered to be associated with impairment of executive function and cognitive control, the present finding suggests that lesion location may have complex associations with treatment-related cognitive changes after stroke. The positive contribution of frontal lobe lesion should therefore be interpreted as a model-derived association rather than evidence that frontal injury directly promotes cognitive recovery. This finding underscores the value of lesion localization in individualized cognitive prognostic assessment.

Finally, diabetes was the only metabolic vascular risk factor retained in the final model. Previous studies have linked diabetes to cognitive decline and impaired daily functioning (49, 50). Diabetes may impair neuroplasticity and cognitive recovery through multiple mechanisms, including aggravation of cerebral small vessel disease, promotion of chronic inflammation, enhancement of oxidative stress, and disruption of cerebral perfusion (51–53). The fact that diabetes was stably retained across multiple feature selection methods suggests that it plays an important role not only in stroke risk but also in post-stroke cognitive recovery. SHAP results indicated that diabetes generally tended to reduce the predicted probability of cognitive improvement, implying that abnormal glucose metabolism may adversely affect cognitive recovery after SCNT-PR. Therefore, in PSCI-MCI patients with diabetes, greater attention to glycemic control and metabolic management may be needed when implementing SCNT-PR and other interventions to optimize the environment for cognitive recovery.

Importantly, the seven retained variables covered multiple clinical dimensions, including baseline cognitive status (corrected MoCA score), cognitive reserve (years of education), stroke severity (NIHSS total score), functional status (ADL score), demographic background (age), lesion localization (frontal lobe lesion), and metabolic risk (diabetes). This indicates that cognitive improvement after treatment in PSCI-MCI is the result of multiple interacting factors and also highlights the value of machine learning in integrating complex clinical information. Compared with models based on single predictors or limited-variable approaches, the present machine learning framework provided an integrated method for combining multiple clinical dimensions. However, given that logistic regression achieved comparable discrimination, the advantage of the machine learning approach should be interpreted mainly in terms of its ability to model complex nonlinear relationships rather than a definitive superiority over conventional regression methods.

Several limitations should be acknowledged. First, this was a single-center retrospective study using a complete-case design, which may reduce sample representativeness. Although predictor-level missingness was assessed and reported transparently, the mechanisms underlying missing data could not be fully determined retrospectively, and missing-not-at-random patterns cannot be completely excluded. Second, the 13-year study period may have introduced temporal heterogeneity due to changes in stroke management, rehabilitation practice, and diagnostic procedures. However, calendar-year information was not retained in the final analytical dataset, preventing formal temporal sensitivity analyses. Third, therapist-level information was unavailable, and potential operator variability could not be assessed. Fourth, although LASSO regression and the Boruta algorithm were used for feature selection, bootstrap-based stability analysis was not performed. Fifth, the outcome was based on 1-month changes in corrected MoCA scores, limiting assessment of long-term cognitive trajectories. In addition, some potentially relevant factors, such as rehabilitation intensity and social support, were not captured. Finally, although SHAP improved model interpretability, the model has not yet been implemented as a clinical tool and should support rather than replace clinical judgment. Future prospective multicenter studies with temporal validation, external validation, and standardized data collection are warranted to further evaluate model robustness and generalizability.

In conclusion, the LightGBM-based model developed in this study showed acceptable predictive performance in predicting cognitive improvement after 1 month of SCNT-PR in patients with PSCI-MCI. Baseline corrected MoCA score, years of education, NIHSS total score, ADL score, age, frontal lobe lesion, and diabetes were identified as the most important predictive features. This model may facilitate early estimation of the likelihood of cognitive improvement following SCNT-PR and provide support individualized clinical assessment and patient stratification in PSCI-MCI. Future prospective multicenter studies are warranted to further validate the clinical applicability and generalizability of the proposed model.

## Data Availability

The original contributions presented in the study are included in the article/Supplementary material, further inquiries can be directed to the corresponding author.

## Glossary

PSCI: post-stroke cognitive impairment
PSCI-MCI: post-stroke mild cognitive impairment
SCNT-PR: scalp cluster needling therapy with prolonged needle retention
ADL score: Barthel Index score for activities of daily living
MoCA: Montreal Cognitive Assessment
MMSE: mini-mental state examination
Fazekas-pvh: Fazekas periventricular hyperintensity
Fazekas-dwmh: Fazekas deep white matter hyperintensity
mRS: modified Rankin Scale
NIHSS total score: National Institutes of Health Stroke Scale total score
SDS: Self-Rating Depression Scale
PSQI: Pittsburgh Sleep Quality Index
LDL-C: low-density lipoprotein cholesterol
HDL-C: high-density lipoprotein cholesterol
INR: international normalized ratio
LASSO: least absolute shrinkage and selection operator
LR: logistic regression
RF: random forest
SVM: support vector machine
ANN: artificial neural network
DT: decision tree
XGBoost: extreme gradient boosting
ROC: receiver operating characteristic
AUC: area under the curve
DCA: decision curve analysis
SHAP: Shapley Additive Explanations
SD: standard deviation
IQR: interquartile range
LightGBM: light gradient boosting machine
