# Supplementary material for: A machine learning model for predicting cognitive improvement after scalp cluster needling therapy in patients with post-stroke mild cognitive impairment
Source: Front Neurol. 2026 Oct 9;17:1884682. doi: 10.3389/fneur.2026.1884682 (PMC13690542; doi:10.3389/fneur.2026.1884682)
Supplement: Supplementary file 1 [file Data_Sheet_1.ZIP › Supplementary/Supplementary Table.docx]

Supplementary Table 1. Logistic regression analysis using MoCA ≥4 points as the outcome definition.

| **Various** | **β coefficient** | **Odds ratio(95%CI)** | ***P*** |
| --- | --- | --- | --- |
| moca_pre | -0.328 | 0.72(0.65–0.80) | <0.001 |
| edu_years | 0.062 | 1.06(1.03–1.10) | <0.001 |
| adl | 0.041 | 1.04(1.02–1.07) | <0.001 |
| nihss_total | -0.206 | 0.81(0.71–0.94) | 0.005 |
| lesion_frontal | 0.628 | 1.87(1.27–2.77) | 0.002 |
| diabetes | -0.336 | 0.71(0.46–1.10) | 0.126 |
| age | 0.01 | 1.01(0.99–1.03) | 0.252 |

Multivariable logistic regression analysis was performed using a stricter definition of cognitive improvement (MoCA ≥4 points). Results are presented as β coefficients, odds ratios (ORs), 95% confidence intervals (CIs), and P values. The analysis was conducted to evaluate whether the major predictors identified in the primary model remained associated with cognitive improvement under an alternative outcome definition.

Supplementary Table 2. Optimal hyperparameter configurations of the machine learning models

| **Model** | **Hyperparameter configurations** |
| --- | --- |
| Logistic regression | optimizer = newton; maxiter = 35; tol = 1e-08; ridge_factor = 1e-10; iterations = 6; converged = True |
| Decision Tree | criterion = gini; splitter = random; max_depth = 5; max_features = sqrt; min_samples_split = 10; min_samples_leaf = 1; ccp_alpha = 0.001; random_state = 123 |
| Random Forest | n_estimators = 100; criterion = gini; max_depth = 10; max_features = log2; min_samples_split = 2; min_samples_leaf = 5; bootstrap = True; oob_score = True; random_state = 123 |
| XGBoost | booster = None; objective = binary:logistic; eval_metric = logloss; learning_rate = 0.01; max_depth = 3; min_child_weight = None; subsample = 0.6; colsample_bytree = 1.0; n_estimators = 200; gamma = None; reg_alpha = None; reg_lambda = None; random_state = 123 |
| LightGBM | boosting_type = gbdt; objective = None; learning_rate = 0.1; num_leaves = 31; max_depth = 5; n_estimators = 100; subsample = 0.6; colsample_bytree = 0.6; min_child_samples = 20; min_child_weight = 0.001; reg_alpha = 0.0; reg_lambda = 0.0; random_state = 123 |
| Support Vector Machine | C = 0.1; kernel = linear; degree = 2; gamma = scale; shrinking = True; probability = True; tol = 0.001; decision_function_shape = ovr; random_state = 123 |
| Artificial Neural Network | hidden_layer_sizes = (10, 10); activation = logistic; solver = adam; alpha = 0.001; learning_rate = constant; learning_rate_init = 0.001; max_iter = 500; early_stopping = False; shuffle = True; random_state = 123 |

Supplementary Table 3. TRIPOD+AI checklist for reporting the machine learning prediction model.

| **TRIPOD+AI** | **Reporting recommendation** | **Location in revised manuscript** |
| --- | --- | --- |
| Title | Identify the study as a clinical prediction model development and evaluation study, including the target population and predicted outcome | Title |
| Abstract | Provide the study objective, design, participants, predictors, outcome definition, modeling methods, and main findings | Abstract |
| Background and objectives | Clearly describe the clinical problem, rationale, and objective of developing the prediction model | Introduction |
| Study design | Report the study design, setting, and retrospective nature of the study | Methods, Section 2.1 |
| Participants | Describe eligibility criteria, study population, and patient selection process | Methods, Section 2.1; Figure 1 |
| Outcome definition | Define the predicted outcome and measurement method | Methods, Section 2.2 |
| Predictors | Describe candidate predictors, measurement methods, and timing of predictor assessment | Methods, Section 2.3 |
| Sample size | Explain sample size considerations and events-per-variable assessment | Methods, Section 2.7 |
| Missing data | Report missing data handling strategy and rationale for complete-case analysis | Methods, Section 2.7; Figure 1 |
| Model development | Describe model development procedures, including feature selection and machine learning algorithms | Methods, Sections 2.5 and 2.7 |
| Feature selection | Report feature selection methods and selected predictors | Methods, Section 2.5; Results, Section 3.4 |
| Model specification | Provide final model configurations and hyperparameters | Supplementary Table 2 |
| Model performance | Report discrimination, calibration, and clinical utility evaluation methods | Methods, Section 2.5 |
| Model evaluation | Report validation strategy and evaluation cohort characteristics | Methods, Section 2.6 |
| Performance measures | Report AUC, PR-AUC, sensitivity, specificity, PPV, NPV, calibration, and decision curve analysis | Results, Section 3.3; Table 2 |
| Model interpretation | Describe explainability methods and interpretation of important predictors | Methods, Section 2.5; Results, Section 3.4 |
| Sensitivity analyses | Report analyses evaluating robustness under alternative outcome definitions and predictor configurations | Results, Section 3.5; Figure 6; Supplementary Figure 1 |
| Reporting of uncertainty | Provide confidence intervals and appropriate interpretation of model performance | Results, Table 2 |
| Clinical interpretation | Discuss potential clinical implications and appropriate use of the prediction model | Discussion |
| Limitations | Discuss limitations including retrospective design, lack of control group, complete-case analysis, and need for external validation | Discussion |
| Reproducibility | Report software environment, package information, and hyperparameter configurations | Methods, Section 2.7; Supplementary Table 2 |
| Model applicability | Clarify that the model is an auxiliary decision-support tool rather than a standalone clinical decision instrument | Discussion; Conclusion |
| External validation | Discuss the need for future external validation studies | Discussion; Conclusion |

TRIPOD+AI: Transparent Reporting of a multivariable prediction model for Individual Prognosis Or Diagnosis using Artificial Intelligence.

Supplementary Table 4. Threshold optimization analysis of the LightGBM model in the validation cohort.

| **Threshold** | **Accuracy** | **Sensitivity** | **Specificity** | **PPV** | **NPV** | **F1-score** | **Youden index** |
| --- | --- | --- | --- | --- | --- | --- | --- |
| 0.10 | 0.723 | 0.994 | 0.032 | 0.724 | 0.667 | 0.838 | 0.026 |
| 0.15 | 0.728 | 0.969 | 0.111 | 0.736 | 0.583 | 0.836 | 0.080 |
| 0.20 | 0.732 | 0.938 | 0.206 | 0.751 | 0.565 | 0.834 | 0.144 |
| 0.25 | 0.737 | 0.925 | 0.254 | 0.760 | 0.571 | 0.835 | 0.179 |
| 0.30 | 0.723 | 0.901 | 0.270 | 0.759 | 0.515 | 0.824 | 0.170 |
| 0.35 | 0.719 | 0.888 | 0.286 | 0.761 | 0.500 | 0.819 | 0.174 |
| 0.40 | 0.719 | 0.863 | 0.349 | 0.772 | 0.500 | 0.815 | 0.213 |
| 0.45 | 0.723 | 0.845 | 0.413 | 0.786 | 0.510 | 0.814 | 0.257 |
| 0.50 | 0.714 | 0.826 | 0.429 | 0.787 | 0.491 | 0.806 | 0.255 |
| 0.55 | 0.719 | 0.801 | 0.508 | 0.806 | 0.500 | 0.804 | 0.309 |
| 0.60 | 0.723 | 0.783 | 0.571 | 0.824 | 0.507 | 0.803 | 0.354 |
| 0.65 | 0.728 | 0.770 | 0.619 | 0.838 | 0.513 | 0.803 | 0.389 |
| 0.70 | 0.701 | 0.720 | 0.651 | 0.841 | 0.477 | 0.776 | 0.371 |
| 0.75 | 0.683 | 0.689 | 0.667 | 0.841 | 0.457 | 0.758 | 0.356 |
| 0.80 | 0.661 | 0.609 | 0.794 | 0.883 | 0.442 | 0.721 | 0.402 |
| 0.85 | 0.643 | 0.571 | 0.825 | 0.893 | 0.430 | 0.697 | 0.397 |
| 0.90 | 0.616 | 0.503 | 0.905 | 0.931 | 0.416 | 0.653 | 0.408 |

PPV: positive predictive value; NPV: negative predictive value. Positive classification was defined as predicted probability greater than or equal to the evaluated threshold. Youden index was calculated as sensitivity plus specificity minus 1.

Supplementary Table 5. Distribution of missing values among candidate predictors in the initial screening cohort (n=943).

| **Variable** | **Missing values, n (%)** |
| --- | --- |
| Lesion multifocal | 32 (3.4%) |
| Lesion frontal | 7 (0.7%) |
| Lesion temporal | 6 (0.6%) |
| Lesion parietal | 8 (0.8%) |
| Lesion occipital | 12 (1.3%) |
| Lesion thalamus | 3 (0.3%) |
| Lesion basalganglia | 2 (0.2%) |
| Lesion cerebellum | 5 (0.5%) |
| Lesion brainstem | 6 (0.6%) |
| Smoking history | 33 (3.5%) |
| Alcohol history | 32 (3.4%) |
| Prior stroke / TIA | 54 (5.7%) |
| Prior cognitive impairment | 78 (8.3%) |
| Baseline rehab | 89 (9.4%) |
| Chei use baseline | 39 (4.1%) |
| Memantine use baseline | 34 (3.6%) |
| Cerebral metabolic drug use baseline | 50 (5.3%) |
| Brain atrophy | 66 (7.0%) |
| Sleep apnea | 94 (10.0%) |
| Daytime fatigue | 80 (8.5%) |
| Medication compliance | 130 (13.8%) |
| Lesion size grade | 66 (7.0%) |
| Fazekas pvh | 73 (7.7%) |
| Fazekas dwmh | 77 (8.2%) |
| Fazekas total | 71 (7.5%) |
| Occupation type | 85 (9.0%) |
| NIHSS language | 157 (16.6%) |
| NIHSS neglect | 151 (16.0%) |
| NIHSS total score | 77 (8.2%) |
| ADL score | 187 (19.8%) |
| MoCA pre | 187 (19.8%) |
| MMSE pre | 186 (19.7%) |
| SDS standard score | 124 (13.1%) |
| PSQI score | 172 (18.2%) |
| Heart rate | 5 (0.5%) |
| Total cholesterol | 9 (1.0%) |
| LDL-C | 8 (0.8%) |
| Triglycerides | 9 (1.0%) |
| HDL-C | 12 (1.3%) |
| Creatinine | 29 (3.1%) |
| BUN | 27 (2.9%) |
| PT | 32 (3.4%) |
| INR | 32 (3.4%) |
| D-dimer | 43 (4.6%) |
| Prior stroke count | 57 (6.0%) |
